# Use of Aloe Vera Gel as Media to Assess Antimicrobial Activity and Development of Antimicrobial Nanocomposites

**DOI:** 10.3390/ijms25115599

**Published:** 2024-05-21

**Authors:** Erwan Rauwel, Geeta Arya, Kristi Praakle, Protima Rauwel

**Affiliations:** 1 Institute of Veterinary Medicine & Animal Sciences, Kreutzwaldi 62, 51006 Tartu, Estonia; kristi.praakle@emu.ee; 2 Institute of Forestry and Engineering, Kreutzwaldi 56/1, 51006 Tartu, Estonia; geeta.arya@ut.ee (G.A.); protima.rauwel@emu.ee (P.R.)

**Keywords:** antimicrobial properties assessment, methodology, nanocomposites, metal nanoparticles, *Aloe vera*, Aloe Vera gel

## Abstract

Antimicrobial resistance is a menace to public health on a global scale. In this regard, nanomaterials exhibiting antimicrobial properties represent a promising solution. Both metal and metal oxide nanomaterials are suitable candidates, even though their mechanisms of action vary. Multiple antimicrobial mechanisms can occur simultaneously or independently; this includes either direct contact with the pathogens, nanomaterial uptake, oxidative stress, ion release, or any of their combinations. However, due to their specific properties and more particularly fast settling, existing methods to study the antimicrobial properties of nanoparticles have not been specifically adapted in some cases. The development of methodologies that can assess the antimicrobial properties of metallic nanomaterials accurately is necessary. A cost-effective methodology with a straightforward set-up that enables the easy and quick assessment of the antimicrobial properties of metal nanoparticles with high accuracy has been developed. The methodology is also capable of confirming whether the killing mechanism involves ionic diffusion. Finally, Aloe Vera gel showed good properties for use as a medium for the development of antimicrobial ointment.

## 1. Introduction

Antimicrobial resistance (AMR) [1] is one of the major health crises that is a looming global threat. Because antibiotics have always been the preferred treatment for bacterial infections, the intensive and broad-spectrum utilization of antibiotic drugs during the past century has engendered AMR. This problem has now gained amplitude in the public health sector, owing to the deteriorating efficacy of existing antibiotic agents in the face of more resistant microbial strains [2]. The same phenomenon occurs with pathogenic fungi and antifungal resistance [3]. The current situation requires the development of new antimicrobial agents [4,5] against which pathogens like bacteria and fungi cannot adapt. To that end, the engineering of antimicrobial nanomaterials appears to be one of the most promising solutions, due to the various processes underlying the antimicrobial mechanisms that include reactive oxygen species (ROS) production, ion diffusion, nanomaterial uptake, and interaction with the pathogen (cell membrane damage, internalization…). Over the last two decades, nanomaterials applied as antimicrobial agents have been developed rapidly [6,7], more specifically to battle AMR. In particular, bio-synthesized nanoparticles [8] have been intensively investigated in that context, with more focus on bio-synthesized silver metal nanoparticles (AgNPs) [9,10].

Several methods have been developed to assess the antimicrobial properties of nanomaterials. Most of these methods have been adapted from antimicrobial drug assessment methods. These methods include methods based on the utilization of agar disk diffusion, agar well diffusion, agar plug diffusion, the cross-streak method, the poisoned food method, and the agar plate gradient method (Supplementary Figure S1). Other methods based on dilution (broth dilution, agar dilution) or ATP bioluminescence and flow cytofluorometry are also available, but are more expensive and require specialized equipment [11,12,13]. The development of methodologies to assess the antimicrobial properties of nanomaterials has been ongoing for two decades. For example, thin-layer chromatography–bioautography used to assess the antibacterial, antifungal, antitumor and antioxidant properties of compounds [14] was also adapted to assess the antimicrobial properties of nanomaterials [15]. Despite promising progress in the development of nanomaterials that exhibit antimicrobial properties, their implication in clinical translation as a relevant antimicrobial agent is still a challenging issue owing to the high degree of inconsistency and variability of existing experimental methodologies involving nanomaterials, more specifically for non-colloidal metallic nanoparticles [16]. The most commonly applied antimicrobial assessment based on the diffusion method is considered the simplest, most convenient, and most cost-effective. However, the existing methods aim to assess the antimicrobial properties of drugs, and they are difficult to apply to non-colloidal nanoparticles. Their fast sedimentation induces errors during pipetted solution sampling. Additionally, these nanoparticles may also have an affinity with the pipette tip surface, anchoring through electrostatic attachment and inducing a larger error more specifically for low nanoparticle concentrations. For these reasons, it is not practically possible to measure the local concentration of these nanoparticles at the site of action with accuracy [17].

Metal nanoparticles are comparably heavier than non-metal or polymeric nanoparticles, so they experience the predominant gravitational sedimentation force over the Brownian diffusion force and inter-particle forces [18,19]. This gravitational sedimentation force provokes rapid sedimentation, resulting in a concentration gradient in the solution from bottom to top, contrary to a colloidal dispersion. This force is more dominant for nanoparticle sizes larger than 10 nm [20]. The possible natural agglomeration of these nanoparticles allows quicker nanoparticle sedimentation. Subsequently, the diffusion flux and the sedimentation flux behave as opposing forces to each other, and at a given point in time, the sedimentation diffusion achieves its equilibrium. Here, both the fluxes become equal and therefore non-competing, and this point in time is called the settling time for the nanoparticles in that particular solution [19]. The setting time implies that the concentration of these nanoparticles may not be homogeneous in the solution during the antimicrobial tests over time. Although these nanoparticles are very small moieties, their dose–response analysis is essential, as the dose is the primary factor that decides their potential for therapeutics, considering their cytotoxicity [21].

It is possible to assess the antimicrobial properties of engineered nanoparticles using disk diffusion susceptibility tests [22]. The Kirby–Bauer method can be applied, but only in the case of nanomaterials that tend to release toxic and highly diffusive ions or organic molecules (i.e., soluble species) into the cultured growth medium. It is possible to assess the antimicrobial properties of AgNPs because they can release silver ions through dissolution. This method can only assess antimicrobial properties based on ion diffusion rather than the physical interaction of the microorganisms with the nanomaterials. However, the disk diffusion method relies on the amount of solution added to the diffusion disk, which is highly dependent on the homogeneity of the prepared solution. For these reasons, the possible agglomeration of nanoparticles or fast sedimentation of the tested nanomaterial during the preparation of the solution must be avoided.

In this study, conventional methods to assess antimicrobial properties were applied and compare the results obtained with Aloe Vera gel diffusion method. In the case of the well diffusion assay method, a higher quantity of solution is required to fill the well, with a lower accuracy than the disk diffusion assay method. For the drop diffusion assay method, the ion or drug release is combined with direct contact established between the nanomaterial and the microorganism under study in the area where the drop was placed. This area is automatically marked owing to the natural trace left behind by the drop in the Petri dish. All these methods are dependent on the solution quantity that will be pipetted to do the tests.

To calculate the accurate dose-dependent response relation of these nanoparticles against microorganisms, there is a need for a methodology that can surmount or bypass these issues related to the settling dynamics of metal nanoparticles in solution. Therefore, this work describes a methodology based on Aloe Vera gel used as diffusion media that enables a better evaluation of the dose-dependent local concentration relationship of these metal nanoparticles against microorganisms. Aloe Vera gel has been selected because it is primarily composed of water similar to PBS, but it exhibits a certain viscosity that deters the sedimentation of the nanoparticles. The high water content in the gel will enable the diffusion of ions released from the nanoparticles trapped in the gel into the agar. In this way, the methodology allows the identification of antimicrobial property-based on ions release in an easy and cost-effective way.

## 2. Results and Discussion

### 2.1. Characterization of the Nanomaterials

**Figure 1:**
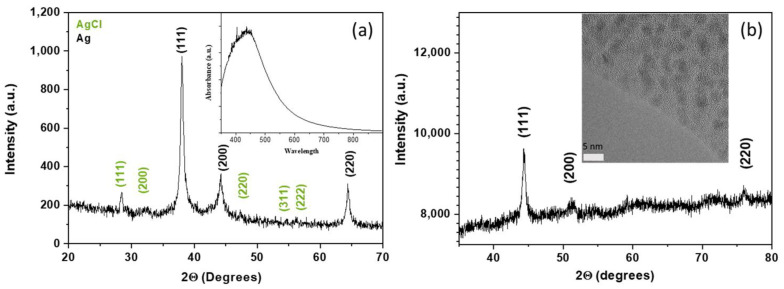
(**a**) XRD pattern of bio-synthesized silver nanoparticles (SAE-AgNPs), inset UV-vis absorption spectrum of SAE-AgNPs sample, and (**b**) XRD pattern of surfactant-free cobalt metal nanoparticles (CoNPs); inset: HRTEM micrograph of cobalt metal nanoparticles.

The XRD pattern of the bio-synthesized AgNPs highlights the presence of cubic Ag metal nanoparticles as the main phase (Figure 1a), with the characteristic diffraction peaks at 38.15°, 44.30°, and 64.52° that correspond to correspond to the (111), (200), and (220) reflections, respectively (ICDD file no. 00-087-0718). The UV-Vis absorption spectrum also confirms the presence of AgNPs with a maximum absorption at 471 nm (inset Figure 1a). The XRD pattern also shows the presence of trace amounts of AgCl secondary phase with X-ray diffraction peaks of very low intensity at 27.88°, 32.26°, 46.25°, 54.85°, and 57.50° that correspond to the (111), (200), (220), (311), and (222) reflections (ICDD file no. 00-001-1013). AgCl is a well-known secondary phase commonly produced when using plant extract-mediated synthesis [23,24]. The presence of AgCl secondary phase in trace amounts is not detrimental for the present study, which focuses on the development of a methodology to assess the antimicrobial properties of metal nanoparticles. In addition, AgCl nanoparticles are known to exhibit antimicrobial properties [25]. The X-ray diffraction pattern of Figure 1b reveals that cobalt metal nanoparticles (CoNPs) exhibit a face-centered cubic (Fm3m) crystal structure (a = 3.5447 Å), and a crystallite average diameter of 3 nm was calculated using the Scherrer equation [26]. HRTEM study confirmed the nanosize of the cobalt metal nanoparticles and their high crystallinity.

### 2.2. Evaluation of Antibacterial Activity

#### 2.2.1. Drop Diffusion Assay

**Figure 2:**
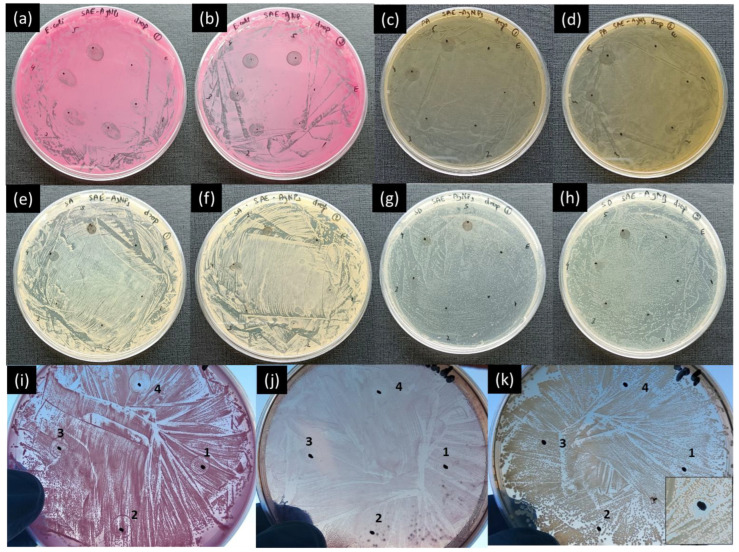
Antimicrobial activities of *Salvia officinalis* silver nanoparticles (SAE-AgNPs) using drop diffusion assay against (**a**,**b**) *E. coli*, (**c**,**d**) *P. aeroginosa*, (**e**,**f**) *S. aureus*, and (**g**,**h**) *S. dysgalactiae*; places marked 1 to 5 correspond to concentrations of 0.1, 0.2, 0.5, 0.8, and 1 mg/mL, respectively, and place E corresponds to pure *Salvia officinalis* aqueous extract. Antimicrobial activities of cobalt metal nanoparticles (CoNPs) using drop diffusion assay against (**i**) *E. coli*, (**j**) *P. aeroginosa*, and (**k**) *S. aureus*. The places marked 1 to 4 correspond to concentrations of 0.001, 0.01, 0.1, and 1 mg/mL, respectively.

The antimicrobial activities of silver nanoparticles are well known; more specifically, when synthesized using plant extracts that also exhibit antimicrobial properties on their own [27]. Five concentrations of Ag nanoparticles (SAE-AgNPs) suspended in PBS were tested against the four different bacterial strains. In Figure 2, the drops marked 1 to 5 correspond to SAE-AgNP concentrations of 0.1, 0.2, 0.5, 0.8, and 1 mg/mL, respectively. The zone marked E (upper right spot) indicates the place where only aqueous plant extract was dropped, serving as the negative control for the experiment.

Figure 2 shows that for all the tested strains (i.e., Gram-negative and Gram-positive), there is an increase in the inhibition area with the increase in SAE-AgNP concentration in PBS (Table 1). It highlights that SAE-AgNPs exhibit high antimicrobial properties against multiple bacterial strains, therefore possessing a large bactericide spectrum. Plant extract control tests show that *Salvia officinalis* extract alone does not exhibit clear antimicrobial properties (indicated as E in the Petri dishes). The comparison of the antimicrobial tests performed against the *Escherichia coli* suggests that even though the tests were conducted the same day with the same prepared solutions and inoculum, differences in the inhibition area are nevertheless visible (Figure 2a,b). Even though the nanoparticles were agitated with a vortex mixture each time before pipetting, these differences are probably due to an error induced during the sampling through the quick settling tendency of the nanoparticles. In the case of *Pseudomonas aeruginosa*, the spot marked 3 (0.5 mg/mL) in the second Petri dish also does not show clear antimicrobial properties, contrary to the spots marked 1 and 2 with lower concentrations (Figure 2d). In the case of *Streptococcus dysgalactiae*, the first test in Figure 2g shows that antimicrobial properties are visible from a nanoparticle concentration of 0.5 mg/mL (spot marked 3). However, the second test (Figure 2h) shows antimicrobial properties for all concentrations, which highlights an inconstancy of this method for the same batch of tests. In the case of *Staphylococcus aureus*, for all tests, only concentrations from 0.8 mg/mL showed bacterial growth inhibition. These antimicrobial tests using the drop diffusion assay were used as a database to be compared with other available methods.

Similar drop diffusion tests were performed with surfactant-free cobalt metal nanoparticles (CoNPs) against *E. coli.* The first tests were performed with concentrations of 0.2 and 0.4 mg/mL without preliminary agitation of the Eppendorf containing the prepared PBS solution with the vortex mixture and with a small quantity of powder (~0.1 mg) directly in the Petri dish (Supplementary Figure S2). The small drop of powder (~0.1 mg) showed clear inhibition zones that need further confirmation. Drop diffusion assays against *E. coli*, *P. aeroginosa*, and *S. aureus* were performed with CoNP concentrations of 0.001 mg/mL, 0.01 mg/mL, 0.1 mg/mL, and 1 mg/mL. The solutions were carefully prepared using the vortex mixture each time before pipetting. Figure 2i–k show the antimicrobial test results against the three bacterial strains ((2i) *E. coli*, (2j) *P. aeruginosa*, (2k) *S. aureus*), and inhibition areas of different diameters for each strain are visible at 1 mg/mL in all the tests. However, similarly to SAE-AgNPs, the antimicrobial test results were not fully reproducible (Supplementary Figure S2) due to the metallic nature of the nanoparticles and the absence of organic coating on CoNPs that would have generated a colloidal solution.

#### 2.2.2. Well Diffusion Assay

**Figure 3:**
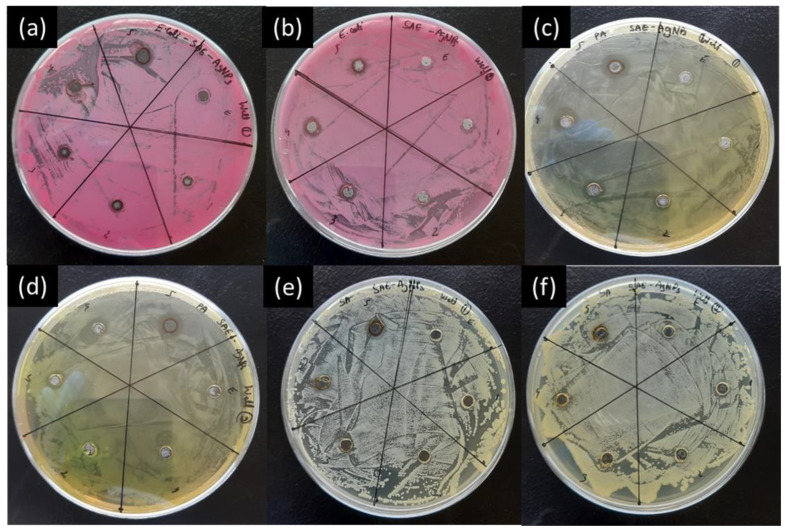
Antimicrobial activity study of SAE-AgNPs using well diffusion assay against (**a**,**b**) *E. coli*, (**c**,**d**) *P. aeroginosa*, and (**e**,**f**) *S. aureus*; places marked 1 to 5 correspond to concentrations of 0.1, 0.2, 0.5, 0.8, and 1 mg/mL, respectively, and place E corresponds to the control test with only *Salvia officinalis* aqueous extract.

A well diffusion assay was applied for comparison with the other conventional diffusion methods and the Aloe Vera gel diffusion assay. Figure 3 shows similar trends with an increase in the inhibition area with the increase in SAE-AgNP concentrations in PBS. However, the tests highlight differences in inhibition areas with the same bacterial strain, with larger inhibition areas for lower nanoparticle concentrations, or changes in inhibition area shape (i.e., other than circular). In the case of tests against *E. coli* (Figure 3a), the SAE-AgNP concentration of 0.8 mg/mL does not show an inhibition area compared to the concentrations of 0.5 and 1 mg/mL, which exhibit antimicrobial properties. In the case of *P. areruginosa* (Figure 3c), a regular increase in the inhibition area with the increase in SAE-AgNP concentration is visible, but the shape is irregular for 0.8 mg/L. A similar trend is visible in Figure 3d, but the shape of the inhibition area is oblong for 1 mg/L. This shows a lack of regularity in the assay that can mainly be attributed to pipetting and the nature of metallic nanomaterials. In the case of Gram-positive bacteria, the well diffusion assay did not show any antimicrobial properties (Figure 3e,f). The well diffusion assay shows that SAE-AgNPs only exhibit antimicrobial properties against Gram-negative bacteria. However, the drop diffusion assay highlighted clear antimicrobial properties against all strains from SAE-AgNP concentrations of 0.8 mg/L (Figure 2). These results demonstrate that the well diffusion assay method does not appear suitable to assess the antimicrobial properties of metal nanoparticles, but is rather more adapted for testing drugs [28].

#### 2.2.3. Disk Diffusion Assay

**Figure 4:**
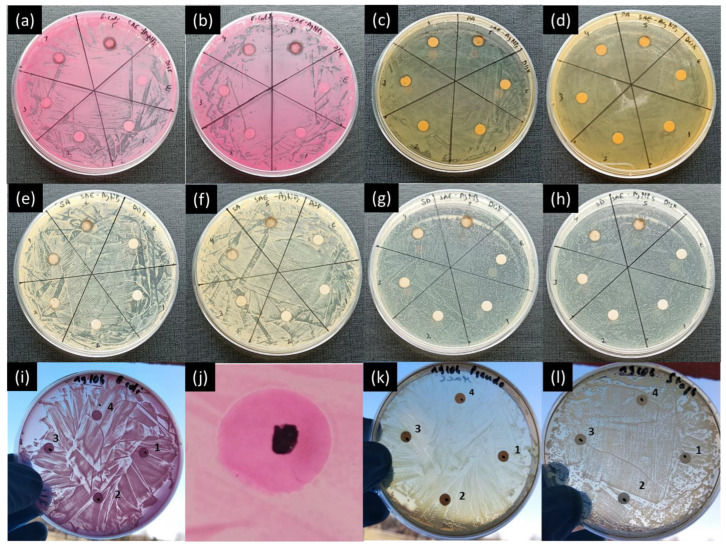
Antimicrobial activity study of SAE-AgNPs using disk diffusion assay against (**a**,**b**) *E. coli*, (**c**,**d**) *P. aeruginosa*, (**e**,**f**) *S. aureus*, and (**g**,**h**) *S. dysgalactiae*; places marked 1 to 5 correspond to concentrations of 0.1, 0.2, 0.5, 0.8, and 1 mg/mL, respectively, and place E corresponds to the control test with only *Salvia officinalis* aqueous extract. Antimicrobial activity study of CoNPs using disk diffusion assay against (**i**,**j**) *E. coli*, (**k**) *P. aeruginosa*, and (**l**) *S. aureus*. The places marked 1 to 4 correspond to concentrations of 0.001, 0.01, 0.1, and 1 mg/mL, respectively.

The disk diffusion assay method was also applied to both the SAE-AgNP and CoNP antimicrobial property assessments. Figure 4 show the results of the disk diffusion method applied to SAE-AgNPs against *E. coli*, *P. aeruginosa*, *S. aureus*, and *S. dysgalactiae*. The disks identified with numbers 1 to 5 correspond to a dose of 2 μL of PBS with concentrations of 0.1 mg/mL, 0.2 mg/mL, 0.5 mg/mL, 0.8 mg/mL, and 1 mg/mL on the disk, respectively, and disk E represents the negative control on which only *Salvia officinalis* aqueous extract was dropped. While some concentrations exhibit slight inhibition zones around the disks, not all the plates show a proper zone of inhibition with SAE-AgNPs against *E. coli* and *P. aeruginosa* (Figure 4a–d). For *E. coli*, similarly to the drop diffusion assay, the disk loaded with a solution of lower concentration (0.2 mg/mL) shows a larger inhibition area than the disk loaded with a solution of higher concentration (0.5 mg/mL) (Figure 4a). As mentioned earlier, this undesired effect could also be due to the settling behavior of the nanoparticles. In the case of *P. aeruginosa*, all concentrations show an inhibition area that increases with an increase in concentration (Figure 4c,d). However, in the case of the drop diffusion assay, the lower concentrations did not show antimicrobial properties (Figure 3c,d), which again highlights a discrepancy between the assessment methods. For *S. aureus* and *S. dysgalactiae*, the disk diffusion assays show antimicrobial properties for the highest concentrations, with a color change around the disk. For the lowest concentrations, the results appear to be less conclusive, but small inhibition zones are visible for 0.2 mg/mL and 0.5 mg/mL (Figure 4e–h). The disk diffusion assay gives more reliable information about the antimicrobial properties than the well diffusion assay. However, the inhibition zone is limited to the circumference of the disk, which makes it more difficult to assess the effect of the concentration on the antimicrobial properties.

In the case of CoNPs, inhibition zones around the disks are visible for *E. coli*, *P. aeruginosa*, and *S. aureus* (Figure 4i–l) for all CoNP concentrations tested (i.e., 0.001, 0.01, 0.1, and 1 mg/mL). However, the drop diffusion assays performed with the same concentrations did not show inhibition areas for the lowest concentrations, and the highest concentrations showed irregularly shaped inhibition areas. The disk diffusion assay seems more suited to testing the antimicrobial properties of CoNPs, but it cannot assess the efficiency as a function of the concentration due to the limited diffusion around the disk. It should be pointed out that for both SAE-AgNPs and CoNPs, the nature of the disk itself (i.e., paper/cellulose) may prevent the appropriate diffusion of the metallic ions into the agar plates.

#### 2.2.4. Gel Diffusion Assay

Multiple studies have been performed to combine *Aloe vera* with nanomaterials [29]. There are a few reports combining Aloe Vera gel and nanomaterials like silver nanoparticles for wound healing [30]. However, none of these studies consider using *Aloe vera* as a medium to assess the antimicrobial properties of metallic nanomaterials. Another major advantage of the gel diffusion method is that only a precision balance is required to mix the engineered nanomaterials with Aloe Vera gel (see Materials and Methods). The gel mixture is then immediately transferred into the 1 mL micro-syringe that enables precisely calibrating a drop of 20 µL of the gel mixture in the Petri dish to ensure the best reproducibility in the tests. In addition, the micro-syringe protects the gel mixture from oxidation and drying. This study shows that the preparation of a homogeneous mixture is a pre-requisite to an accurate assessment of antimicrobial properties. In this study, the antimicrobial properties of Ag nanoparticles synthesized using *Salvia officinalis* plant extract (SAE-AgNPs) and surfactant-free cobalt metal nanoparticles (CoNPs) have been assessed as case examples. These CoNPs were recently investigated and demonstrate potential applications in nanomedicine [26] and water purification [31].

**Figure 5:**
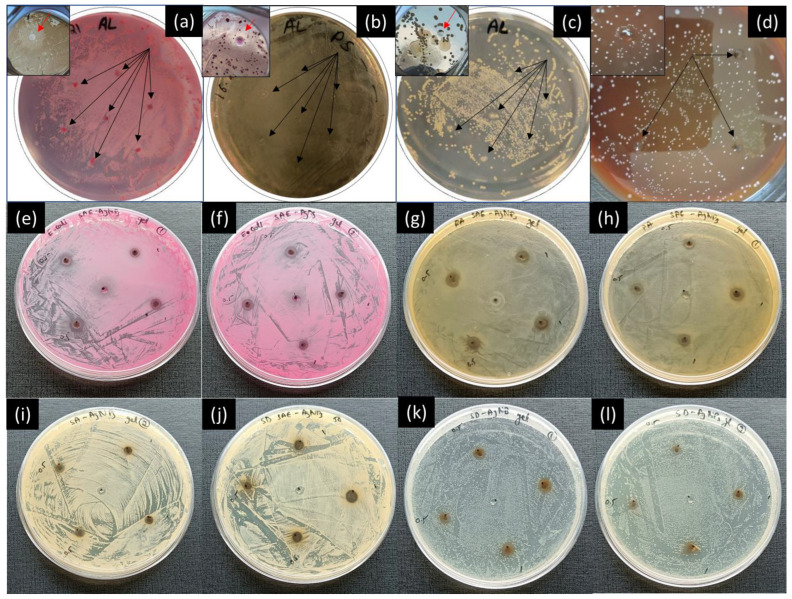
Antimicrobial activities study of pure Aloe Vera gel (20 μL) assay against (**a**) *E. coli*, (**b**) *P. aeroginosa*, (**c**) *S. aureus*, and (**d**) *S. dysgalactiae*; insets are magnified, highlighting the absence of an inhibitory area. Antimicrobial activity study of *Salvia officinalis* silver nanoparticles (SAE-AgNPs) mixed with Aloe Vera gel (20 μL) against (**e**,**f**) *E. coli*, (**g**,**h**) *P. aeruginosa*, (**i**,**j**) *S. aureus*, and (**k**,**l**) *S. dysgalactiae*; places marked 0.5 and 1 correspond to concentrations of 0.5%wt. and 1%wt. The drop visible in the center is pure Aloe Vera gel used as a negative control that does not exhibit any inhibition zone.

The pure Aloe Vera gel was tested against all bacterial strains used in this study to confirm the absence of antimicrobial properties against these strains. Drops of 20 μL of gel were placed in Petri dishes preliminarily inoculated with each bacterial culture (See Materials and Methods). In Figure 5a–d, no antimicrobial properties are visible for all tested bacteria. All the insets highlight the absence of inhibition zones and the presence of bacterial colonies that are growing in the vicinity and on the surface of the Aloe Vera gel drops. The control tests showed that Aloe Vera gel does not exhibit antimicrobial properties against the tested strains, which makes it a suitable diffusion gel.

To assess the validity of the method and compare it with the results obtained from the other assays, SAE-AgNPs were mixed with Aloe Vera gel, considering the weight ratios of 0.5 wt% and 1 wt%. Two drops of each concentration were added to the Petri dishes (Figure 5e–l), and one drop of pure Aloe Vera gel was also added to the center as a negative control.

Figure 5e–l shows the antimicrobial assay results of the gel drop method of SAE-AgNPs against *E. coli*, *P. aeruginosa*, *S. aureus*, and *S. dysgalactiae*. In all the Petri dishes, zones of inhibition are clearly visible against all bacterial strains for both 0.5 wt% and 1 wt%. The pure Aloe Vera gel drops in the center of the plate do not show antimicrobial properties. However, for *E. coli*, a lower density of bacterial colonies was sometimes visible around the Aloe Vera gel compared to the colony density in the whole Petri dish. This shows that Aloe Vera gel sometimes affected *E. coli* development by reducing the number of colonies without preventing bacterial development and fully inhibiting bacterial growth around the gel drop (Figure 5e,f). The inhibition areas were measured as the distance between the gel and the first grown colonies (radius) to promote accuracy in the measurement. The comparison of the inhibition distance between Gram-negative and Gram-positive bacteria indicates that for Gram-positive bacteria, the inhibition radius increases with the increase in the SAE-AgNP quantity in the gel (Table 2). However, for Gram-negative bacteria, the inhibition radius remains the same for both 0.5 wt% and 1 wt% ratio. This highlights that this assessment method is based on nanoparticle concentrations and not diffusivity. Additionally, similar to the other assessment methods, the SAE-AgNPs exhibit higher antimicrobial properties against Gram-negative than Gram-positive bacteria, with the most sensitive bacteria being *P. aeruginosa*.

**Figure 6:**
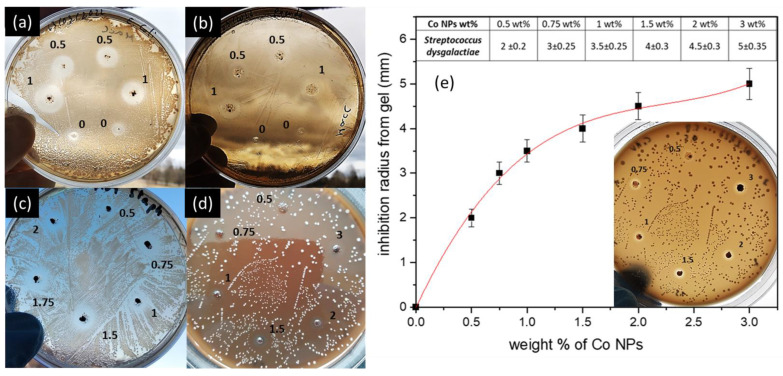
Antimicrobial activities of cobalt metal nanoparticles (CoNPs) mixed with Aloe Vera gel against (**a**) *E. coli*, (**b**) *P. aeroginosa*, and (**c**) *S. aureus*; places marked 0.5, 0.75, 1, 1.5, 1.75, and 2 correspond to concentrations of 0.5, 0.75, 1, 1.5, 1.75, and 2 wt% in the drop of 20 μL of gel added; (**d**) *S. dysgalactiae* places marked 0.5, 0.75, 1, 1.5, 2, and 3 correspond to concentrations of 0.5, 0.75, 1, 1.5, 2, and 3 wt% in the drop of 20 μL of gel added; (**e**) inhibition radius depends on the amount of CoNPs in the Aloe Vera gel from (**d**).

The antimicrobial properties of CoNPs using similar nanoparticle concentrations (0.5 wt% and 1 wt%) were also studied using Aloe Vera gel as a diffusion medium. Figure 6a–c show that CoNPs exhibit antimicrobial properties against all the strains, and the inhibition radius increases directly with the weight ratio (Table 3). The distance almost doubles with the increase from 0.5 wt% to 1 wt%. For the same quantity of nanoparticles in the gel, the inhibition radius is larger for CoNPs than SAE-AgNPs. These inhibition distances are directly comparable because the tests were made in exactly the same conditions, with similar amounts of nanomaterials and the same bacterial strains. From this experiment, it can be concluded that CoNPs exhibit higher antimicrobial activity due to cobalt ion release.

In the case of *S. dysgalactiae*, which is known to be more resistant to antibiotics, CoNP weight ratios ranging from 0.5 wt% to 3 wt% were tested. Figure 6d shows that all concentrations (wt%) show inhibition zones with an inhibition radius that increases with the increase in CoNPs in the Aloe Vera gel. Figure 6e shows the logarithmic increase that exists between the amount of CoNPs in the Aloe Vera gel and the inhibition radius. The increase in radius is clearly visible in Figure 6d and the inset of Figure 6e, which highlights the concentration dependence with the inhibition area. Because the Aloe Vera gel drops added in the Petri dishes were precisely measured, the higher radius can be directly related to the CoNP content in the gel, which directly links the dependency of the antibacterial efficiency to the concentration.

### 2.3. Comparison between Drop Diffusion Assay and Gel Diffusion Assay

#### 2.3.1. Antimicrobial Tests against Bacterial Strains: Silver Nanoparticles

**Figure 7:**
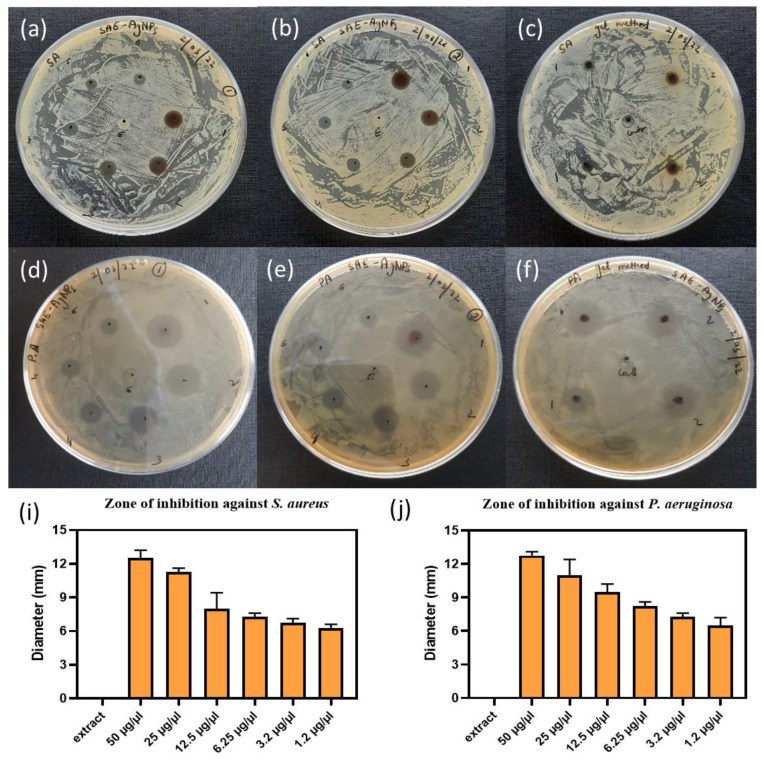
Comparison of antimicrobial activities of *Salvia officinalis* silver nanoparticles (SAE-AgNPs) against *S. aureus* (**a**) and (**b**) drop diffusion assay, (**c**) Aloe Vera gel diffusion assay against *S. aureus*, and (**d**) and (**e**) drop diffusion assay, (**f**) Aloe Vera gel diffusion assay against *P. aeruginosa*; (**i**) average zone of inhibition (D, diameter, mm) for (**a**,**b**), and (**j**) average zone of inhibition for (**d**,**e**). The places marked from 1 to 6 and E in the drop diffusion assay correspond to concentrations of 50, 25, 12.5, 6.25, 3.2, and 1.2 mg/mL and only plant extract, respectively; the places marked 1, 2, and “gel” (center of the Petri dish) in Aloe Vera gel diffusion assays correspond to concentrations of 0.5 wt%, 1 wt%, and pure Aloe Vera gel, respectively.

For comparison, the antimicrobial properties of SAE-AgNPs against *P. aeruginosa* (Gram-negative) and *S. aureus* (Gram-positive) (Figure 7) were assessed by both drop diffusion and Aloe Vera gel diffusion assays. In order to obtain a relevant comparison, the concentration of SAE-AgNPs used in the drop diffusion assay was increased and varied from 1.2 to 50 mg/mL by systematically halving the preceding higher concentration, similar to an arithmetic series. In the case of the *Aloe Vera* gel diffusion method, weight ratios of 0.5 wt% and 1 wt% were prepared.

For the drop diffusion assay, the antimicrobial properties of SAE-AgNPs are confirmed using higher concentrations, and a regular increase in the inhibition zone is visible with an increase in the SAE-AgNP concentration (Figure 7a,b,d,e). For *S. aureus*, a stabilization of the inhibition zone size at a diameter of 7 mm is observed at a concentration of 25 mg/mL (Figure 7i). This suggests that increasing the nanoparticle concentration to 50 mg/mL does not improve their antibacterial efficiency. In the case of *P. aeruginosa*, the inhibition zone size reaches a maximum diameter of 12 mm for a concentration of 50 mg/mL (Figure 7j). For the Aloe Vera gel diffusion assay, the inhibition radius increases with the increase in SAE-AgNP content in the gel against both *S. aureus* and *P. aeruginosa* (Figure 7c,f). Similar to the drop diffusion assay, the Aloe Vera gel diffusion assay reveals that SAE-AgNPs exhibit higher antimicrobial properties against *P. aeruginosa* than *S. aureus*. This comparison shows that both methods produce similar trends and results, and that the drop diffusion method validates the Aloe Vera gel diffusion assay in the case of SAE-AgNPs. The Aloe Vera gel diffusion assay can then also be applied to assess the antimicrobial properties of silver metal nanoparticles and can be extended to other metallic nanomaterials.

#### 2.3.2. Antimicrobial Tests against Fungal Strain: Cobalt Nanoparticles

The Aloe Vera gel assay was also tested with fungi that exhibit a different metabolism than bacteria to validate the method to a broader spectrum of pathogens. *Microsporum canis* is a known dermatophyte that falls under the family *Arthrodermataceae*, and affects pets and cattle [32]. It can be transmitted to humans by contact with a contaminated pet. It was selected due to its resilience and typical cottony growth with either creamy yellowish or brownish color.

**Figure 8:**
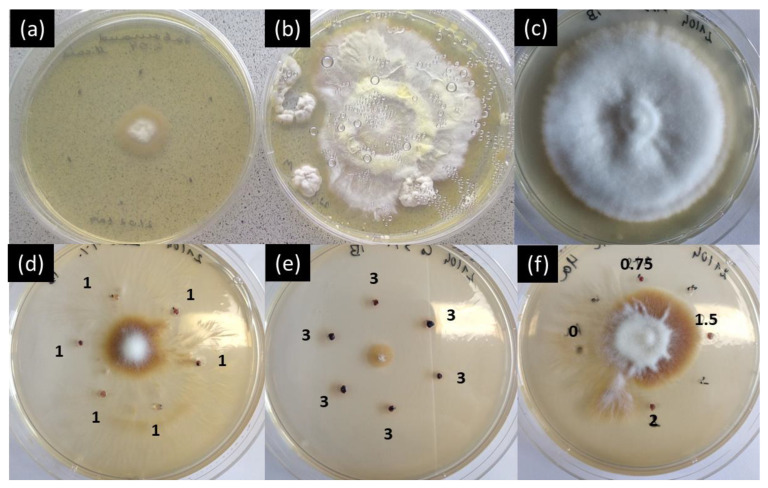
Inhibition area against *M. canis* seeded in the center of the plate (**a**) surrounded by six 10 μL drops (marked with a black dot) of PBS containing 400 mg/L of CoNPs; (**b**) control plate; (**c**) control plate with pure Aloe Vera gel drops; (**d**,**e**) antifungal activity of Aloe Vera gel drops containing weight ratios of 1 wt% and 3 wt% of CoNPs against *M. canis*; (**f**) antifungal activity of CoNPs against *M. canis;* places marked 0, 0.75, 1.5, and 2 correspond to concentrations of pure Aloe Vera gel and 0.75 wt%, 1 wt%, and 2 wt% CoNPs added in 20 μL drops.

Antimicrobial studies using a PBS solution containing 400 mg/L of CoNPs or Aloe Vera gel loaded with CoNPs at weight ratios of 1 wt% and 3 wt% were compared. The tests were performed by adding six drops of PBS (10 μL) or Aloe Vera gel (20 μL) around the seeding place in the center of the Petri dish. In this way, it was possible to assess whether the growth and killing of *M. canis* was due to the nanoparticles. Figure 8a shows inhibition areas all around the six PBS drops (black dots); the *M. canis* in the center could not grow further, contrary to the Petri dish in which only *M. canis* was seeded (Figure 8b). Similar tests were performed with Aloe Vera gel containing 0, 1, and 3 wt% of CoNPs (Figure 8c–e). The tests show that *M. canis* growth is inversely proportional to the nanoparticle concentration. In addition, *M. canis* becomes brown when near the *Aloe Vera* gel dots containing CoNPs. In the absence of CoNPs in Aloe Vera gel, *M. canis* grew normally, and remained cottony and white (Figure 8c). The proximity of CoNPs kills *M. canis* through possible ion release from the gel that is proportional to CoNP concentrations in the Aloe Vera gel. For confirmation, a Petri dish containing different concentrations of CoNPs was prepared (0, 0.75, 1, and 2 wt%). Figure 8f highlights a direct relationship between the CoNP concentration in the gel and the inhibition radius. In certain cases, a larger growth of *M. canis* was observed, but it systematically resulted in the death of *M. canis* (brown color) with no further growth, even after one month (Figure 8f). This study confirms that the Aloe Vera gel diffusion assay can also be applied to assess antifungal properties, and a high reproducibility was observed during the experiments. Fungi are known to be more resilient than bacteria and more complicated to treat. The present study revealed the antifungal properties of CoNPs against fungi when combined with Aloe Vera gel. This opens the path for the development of antifungal ointment nanocomposites that needs to be further studied.

## 3. Materials and Methods

### 3.1. Materials

Luria broth and agar, PBS, absolute ethanol, 70% ethanol, Whatman filter paper no.1, cork-borer, 1 mL micro-syringe, inoculation loop, L-spreader, clear organic Aloe Vera gel, the culture inoculum for two Gram-positive and two Gram-negative bacteria, bio-synthesized silver metal nanoparticles, and surfactant-free cobalt metal nanoparticles. Bacterial strains were cultivated in Petri dishes containing MacConkey LAB AGAR (Biomaxima S.A., Lublin, Poland), Columbia agar base (VWR Chemicals, Leuven, Belgium) for *Escherichia coli*, *Pseudomonas aeruginosa*, *Staphylococcus aureus* and *Streptococcus dysgalactiae*. *Microsporum canis* was cultivated in Petri dishes containing Sabouraud Dextrose with Chloramphenicol (Biomaxima, S.A., Lublin, Poland). The experiments only required a weighting balance and incubator. *Microsporum canis* isolates (code: YY06933759 and YY06646371) of human patients were obtained from the laboratory of the Tartu University Hospital (Tartu Ülikooli Kliinikum) for the collection of microbial strains. The study was excluded from review by the Research Ethics Committee of the University of Tartu as the data were fully anonymized and the related patient information was not available to the research team. The study adhered to the tenets of the Declaration of Helsinki. *Escherichia coli*, *Pseudomonas aeruginosa*, *Staphylococcus aureus*, and *Streptococcus dysgalactiae* were obtained from the National Centre for Laboratory Research and Risk Assessment (LABRIS) in Estonia. All bacterial isolates and fungal isolates were identified by MALDI-TOF mass spectrometry and the polymerase chain reaction method, respectively.

### 3.2. Nanomaterials

Bio-synthesized AgNPs were prepared using *Salvia officinalis* aqueous plant extract (SAE). SAE was prepared using 2 g of leaves (Josenea Bio S.L.U.), powdered and then boiled in 100 mL of milli-Q water at 60 °C. The clean, dark brown color filtrate was obtained under vacuumed filtration. The bio-synthesis of AgNPs was performed using 1 mL of SAE with 9 mL of different concentrations of prepared AgNO_3_ solution, including 20 mM and 50 mM, and the reaction was performed at 60 °C. *Salvia officinalis* aqueous extract-bio-synthesized AgNPs (SAE-AgNPs) were then dried at 40 °C. Surfactant-free cobalt metal nanoparticles were of industrial grade and were provided by PRO-1 NANOSolutions OÜ.

### 3.3. Experimental Details

#### 3.3.1. Characterization of the Nanoparticles

X-ray diffraction (XRD) data were collected using a Bruker D8 Discover instrument equipped with a LynxEye detector. Cu (ka1 = 0.154056 nm) radiation selected by a Ge (111) monochromator was used. High-resolution computed X-ray tomography (XRT) was carried out on YXLON FF35 CT. High-resolution transmission electron microscopy (HRTEM) was carried out on a 80–300 FEI Titan, operating at 300 kV, using a point-to-point resolution of 1.4 Å.

#### 3.3.2. Antimicrobial Properties Assay

To validate the methodology, the antimicrobial properties of the bio-synthesized silver nanoparticles and surfactant-free cobalt metal nanoparticles were studied using different diffusion assay methods. The results of the antimicrobial study using the conventional drop diffusion assay were compared with the results obtained from the utilization of Aloe Vera gel as the diffusion medium. The study was performed against two Gram-negative bacteria (*Escherichia coli*, *Pseudomonas aeruginosa*), two Gram-positive bacteria (*Staphylococcus aureus, Streptococcus dysgalactiae*), and one fungus (*Microsporum canis*). The primary culture inoculum of each bacterium was prepared one day before the experiment. During the antimicrobial tests, before the addition of the medium containing the nanoparticles (PBS or gel), the culture inoculum of each bacterium was evenly spread on the agar plates. The exact inoculum size was determined before the experiment was performed. The optimal inoculum size for plating was found to be 10^6^ microbial cells per milliliter. After addition, the plates were then incubated at 37 °C. The inhibition zones were measured after 24 h and compared. During the antifungal tests, before the addition of the medium containing the nanoparticles (PBS or gel), the inoculum with *M. canis* was placed in the center of the plate. The plates were then incubated for three weeks at 30 °C. The plates were visually assessed every week for up to three weeks, and the macroscopic morphology was observed by eye. The experiments were performed in triplicate.

- Drop diffusion assay

A drop diffusion assay was used to assess the antimicrobial properties of the bio-synthesized silver nanoparticles and surfactant-free cobalt metal nanoparticles. For the antibacterial test, 2 µL of PBS solution containing different concentrations of bio-synthesized silver nanoparticles ranging from 0.1 to 1 mg/mL were dropped in the agar plates, and the plates were then incubated at 37 °C. The inhibition zones were measured after 24 h and compared. In the case of surfactant-free cobalt nanoparticles, concentrations ranging from 0.001 to 1 mg/mL were studied. The experiments were performed in triplicate. For antifungal tests, only surfactant-free cobalt metal nanoparticles were tested, and 10 µL of PBS solution drops containing 400 mg/L of CoNPs was added to the plates at 6 equidistant specific spots according to a similar pattern in each plate, surrounding the *M. canis* inoculum. The plates were then incubated for three weeks at 30 °C. The plates were visually assessed every week for up to three weeks, and the macroscopic morphology was observed by eye. The experiments were performed in triplicate.

- Well diffusion assay

The well diffusion assay was performed on bio-synthesized silver nanoparticles (SAE-AgNPs) to compare the inhibition zone with the drop assays to achieve a clearer comparison with the methodology based on the Aloe Vera diffusion gel assay. The culture inoculum of each bacterium was evenly spread on the agar plates and the well was created on the cultured plate with the help of a sterile cork-borer. The wells were then filled with different concentrations of SAE-AgNPs ranging from 0.1 to 1 mg/mL. The plates were then incubated at 37 °C, and the zones of inhibition were measured after 24 h. The experiments were performed in triplicate.

- Disk diffusion assay

The disk diffusion assay is widely used to study the antimicrobial properties of new drugs or nanoparticles; for this reason, this assay was also performed on the bio-synthesized silver nanoparticles (SAE-AgNPs) and surfactant-free cobalt metal nanoparticles. During the antimicrobial tests, before the addition of disk diffusion and their impregnation, a culture inoculum of each bacterium was evenly spread on the agar plates. The autoclaved disks prepared from Whatman filter paper were placed on the cultured plate properly distant from each other. The disks were then impregnated with 2 µL of PBS solution containing different concentrations of nanoparticles ranging from 0.1 to 1 mg/mL. The plates were then incubated at 37 °C, and the inhibition zones were measured after 24 h. In the case of the surfactant-free cobalt nanoparticles, concentrations of 0.001 to 1 mg/mL were studied. The experiments were performed in triplicate.

- Aloe Vera gel diffusion assay

**Figure 9:**
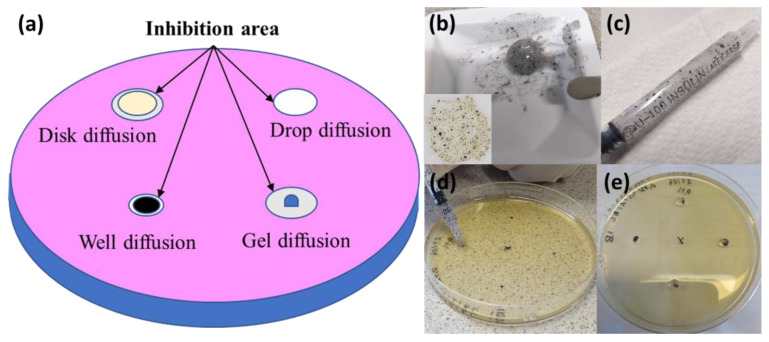
(**a**) Different diffusion assay methods applied in this study; (**b**) mixture of Aloe Vera gel and cobalt metal nanoparticles, inset: image from optical microscope; (**c**) syringe filled with Aloe Vera gel mixture; (**d**) addition of 20 µL of gel mixture in inoculated Petri dish; and (**e**) inoculated Petri dish after the addition of four Aloe Vera gel drops for antimicrobial assessment.

The diffusion testing method was modified by using the agar plate method and impregnating the various metal nanoparticles in a gel-based formulation. The Aloe Vera gel assessment method was then compared with other conventional methods, i.e., disk diffusion, drop diffusion, and well diffusion (Figure 9a). Due to the fact that the Aloe Vera gel was composed of 98.5% of water, which makes it an aqueous medium, the hypothesis was that this method would work on the principle of ion diffusion. The nanoparticles were not in direct contact with the bacterial culture because the Aloe Vera gel exhibited a higher density than the aqueous solution, and the metallic nanomaterials were confined in the gel and could not migrate in the Petri dish. For the tests, a gel that was primarily composed of water similar to PBS was selected, but with a certain viscosity that deterred the sedimentation of the nanoparticles. For these reasons, the Aloe Vera gel was selected, because *Aloe vera* is a natural non-toxic hydrogel that is mainly composed of 98.5% water [33]. Due to its known regenerative, antioxidant, and moisturizing properties, *Aloe vera* has been intensively investigated for its applications in cosmetics and biomedical applications in gel form [34]. The antimicrobial properties of *Aloe vera* have been investigated, and many different reports have been published. A study of *Aloe vera* juice showed that it can exhibit antimicrobial properties only against specific bacteria [35], and no antimicrobial properties were found against the bacteria tested in this manuscript, nor against fungi like *Candida albicans*. In another study, *Aloe vera* extract in ethanol showed similar antimicrobial properties to sodium hypochlorite; however, the authors did not test pure *Aloe vera* extract [36]. In another study, the antimicrobial properties of *Aloe vera* pulp mixed in one liter of 2% dimethyl sulfoxide (DMSO) were investigated using a microbroth dilution method [37], and showed a Minimum Inhibitory Concentration of 50% (MIC_50_) against *Pseudomonas aeruginosa* for an *Aloe vera* concentration of 200 μg/mL. The authors conclude that *Aloe vera* pulp could be active against *P. aeruginosa* at various concentrations. However, other studies have shown that 2% DMSO also induces the growth inhibition of *Pseudomonas aeruginosa* [38]. A more recent study showed that *Aloe barbadensis miller* extract exhibits antimicrobial properties against *E. coli*, *Shigella spp.*, *Salmonella spp.,* and *S. aureus* [39]. Because of the different results and reports found in the literature, the antimicrobial properties of the pulp from the *Aloe vera arborescent* plant and the Aloe Vera gel used in this study were first tested against all selected bacteria and fungi to confirm the absence of antimicrobial properties of the gel alone.

To compare with other diffusion assay methods, the antimicrobial properties of the bio-synthesized silver nanoparticles and surfactant-free cobalt metal nanoparticles were tested using the gel diffusion assay method. The nanomaterials were first crushed gently with a mortar and pestle to obtain a fine and homogeneous powder that was mixed with commercially available Aloe Vera gel. The weight ratio was measured using a Kern ABS 220 precision balance (0.1 mg of precision) to exactly measure the weight ratio of nanomaterial/Aloe Vera gel (Figure 9b). Different nanomaterial weight percentage ratios ranging from 0.5 wt% to 3 wt% were prepared. It is very important to mix the nanoparticles properly in the gel to assure the homogenous dispersion of the nanoparticles in the gel because only drops of 20 µL were added in selected areas of the Petri dish. After proper mixing, the gel mixture was immediately transferred into the 1 mL micro-syringe (Figure 9c). The advantage of adding the gel to the syringe immediately is to prevent the oxidation and the drying of the gel under air [40,41]. The mixture of Aloe Vera gel and metal nanoparticles was kept in the syringe or any sealed containers for weeks without drying. The syringe enabled the precise control of the addition of 20 µL of gel to ensure the best reproducibility in the tests (Figure 9d,e). X-ray tomography and optical microscopy studies confirmed the homogeneous distribution of the CoNPs spread in the Aloe Vera gel (Supplementary Figure S3). The first experiments were conducted using Aloe Vera gel from fresh leaves of *Aloe vera arborescent* (Supplementary Figure S4a,b). However, during the preparation of the gel mixture, the Aloe Vera gel from fresh leaves converted into a solution containing lumps, and the resulting mixture was close to the mixture obtained with PBS (drop method). The antimicrobial tests performed were consistent (Supplementary Figure S4c), but the utilization of the Aloe Vera gel from fresh leaves showed similar issues to the PBS drop method, i.e., different inhibition areas for the same concentration. This result is attributed to the fact that no gel could be obtained from mixing with nanoparticles, only a liquid solution. In addition, the Aloe Vera gel from fresh leaves needed to be sterilized, which added a step to the methodology process. For these reasons, the utilization of commercially available Aloe Vera gel was preferred.

For the antibacterial test, 20 μL of different weight ratios of nanoparticles mixed in the Aloe Vera gel were then added to the cultured plate in equal amounts with the help of a micro-syringe (Figure 9d,e). The plates were then incubated at 37 °C, and the zones of inhibition were measured after 24 h. The experiments were performed in triplicate. For the antifungal test, 20 µL of pure Aloe Vera gel or Aloe Vera gel loaded with precisely weighted CoNP quantities were added to the plates at 6 equidistant specific spots according to a similar pattern in each plate, surrounding the *M. canis* inoculum. The plates were then incubated for three weeks at 30 °C. The plates were visually assessed every week for up to three weeks, and the macroscopic morphology was observed by eye. The experiments were performed in triplicate.

## 4. Conclusions

Different antimicrobial assessment methods were tested and compared using the same batch of metal nanoparticles. Due to the lack of reproducibility in the assessment of the antimicrobial properties of metal nanoparticles using established methods, more specifically if they are surfactant-free or uncoated with organic moieties, a methodology based on Aloe Vera gel is investigated. Aloe Vera diffusion gel enables the assessment of the antimicrobial properties of metallic nanomaterials with reliability, reproducibility, and accuracy. Antimicrobial mechanisms of metallic nanomaterials include ionic diffusion, and the most suitable way to assess this property is by using a viscous medium, such as a gel that promotes ionic diffusion. This technique therefore allowed us to introduce a methodology based on Aloe Vera gel for testing the antimicrobial properties of metal nanoparticles and bypass the limitations of the existing methodologies due to fast settling in solution, more specifically for low concentrations.

The direct applicability of a method involves simplicity, reliability, and cost-effectiveness which enables its large-scale and immediate use by the scientific community in routine testing. Aloe Vera gel was selected for its composition (98.5% of water), its availability, and its low cost. With the gel drop method, it is possible to actually accurately measure the concentrations of nanoparticles (%wt) and avoid errors due to the rapid sedimentation of metallic nanomaterials, more specifically if they are not colloidal. The method requires first assessing the antimicrobial properties of the pure Aloe Vera gel itself (or any gel), and proceeding systematically with control tests. This methodology can also be extended to other metallic nanomaterials that were not tested in this study, such as copper, nickel, and gold. The complete antimicrobial study of CoNPs is presently under investigation using Aloe Vera gel assays, and will be the subject of a future scientific report to follow up this study. Conventional methods were unable to give reliable results. All research groups in the field can test this method and apply it to their research, allowing them to rapidly evaluate the antimicrobial properties of the metallic nanomaterials under investigation. This method can also be used to identify if the killing mechanism is based on ionic diffusion. This must be further tested to be extended to metal oxide nanoparticles in which the antimicrobial mechanism is either fully or partly based on ionic diffusion and involves ROS production through photoexcitation.

Finally, the combination of Aloe Vera gel and nanoparticles (metal oxide and metal) should be further investigated as a means to develop antimicrobial ointment to battle infection and antimicrobial resistance.

## Figures and Tables

**Table 1:** Inhibition radius of *Salvia officinalis* silver nanoparticles (SAE-AgNPs) for different concentrations against four bacterial strains.

SAE-AgNPs (mg/mL)	Inhibition Radius (From Center of the Drop Diffusion Method) in mm			
	*Escherichia coli*	*Pseudomonas aeruginosa*	*Staphylococcus aureus*	*Streptococcus dysgalactiae*
Extract	0	0	0	0
0.1	0	0	0	0
0.2	8 ± 0.25	6 ± 0.25	0	0
0.5	8 ± 0.15	7 ± 0.25	0	6 ± 0.25
0.8	9 ± 0.2	9 ± 0.2	5 ± 0.15	6 ± 0.1
1	9 ± 0.15	9 ± 0.15	5 ± 0.1	7 ± 0.15

**Table 2:** Inhibition radius of *Salvia officinalis* silver nanoparticles (SAE-AgNPs) for different concentrations against four bacterial strains.

SAE-AgNPs (wt%)	Inhibition Radius (Distance from Gel) in mm			
	*Escherichia coli*	*Pseudomonas aeruginosa*	*Staphylococcus aureus*	*Streptococcus dysgalactiae*
0	0	0	0	0
0.5	2.5 ± 0.25	3.5 ± 0.25	1.15 ± 0.25	1.5 ± 0.25
1	2.5 ± 0.25	3.5 ± 0.25	1.5 ± 0.25	2 ± 0.25

**Table 3:** Inhibition radius of cobalt metal nanoparticles (CoNPs) for different weight ratios with Aloe Vera gel.

CoNPs (wt%)	Inhibition Radius (Distance from Gel) in mm		
	*Escherichia coli*	*Pseudomonas aeruginosa*	*Staphylococcus aureus*
0	0	0	0
0.5	3.0 ± 0.25	2.0 ± 0.25	1.5 ± 0.25
1	4.0 ± 0.25	2.5 ± 0.25	2.5 ± 0.25

## Data Availability

The data presented in this study are available on request from the corresponding author.

## Supplementary Materials

The following supporting information can be downloaded at: https://www.mdpi.com/article/10.3390/ijms25115599/s1.
