# Supplementary material for: Use of Aloe Vera Gel as Media to Assess Antimicrobial Activity and Development of Antimicrobial Nanocomposites
Source: Int J Mol Sci. 2024 May 21;25(11):5599. doi: 10.3390/ijms25115599 (PMC11171552; doi:10.3390/ijms25115599)
Supplement: Supplementary file 1 [file ijms-25-05599-s001.zip › ijms-2991760-supplementary.pdf]

SUPPLEMENTARY INFORMATION

Aloe Vera gel as media to assess the antimicrobial activity and for development of antimicrobial nanocomposites

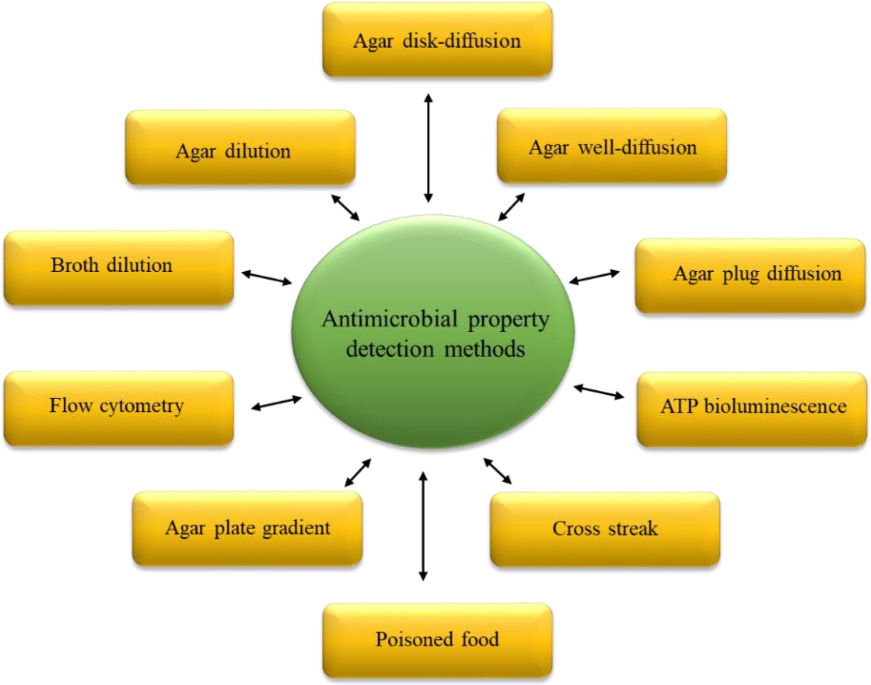

Figure S1. Different methodologies to assess antimicrobial properties.

SI2. Drop diffusion assay on Cobalt metal nanoparticles

Drop diffusion tests were performed with surfactant-free cobalt metal nanoparticles (CoNPs) against *E. coli*. The first tests were performed with concentration of 0.2 and 0.4 mg/mL without preliminary agitation of the Eppendorf containing the prepared PBS solution with the vortex mixture. Only shaking by hand was performed before taking the solution sample with a pipette. Figures S2a, b show the Petri dish used for these tests, and no antimicrobial effect can be explicitly confirmed for a concentration of 0.2 mg/mL (Fig. S2a), but for 1 of the drops containing 0.4

mg/mL (Fig. S2b), an inhibition zone with a diameter of 10 mm is clearly visible on the left. To confirm the probable antimicrobial properties of CoNPs, a small quantity of CoNPs powder (~ 0.1 mg) was directly dropped on the 7 spots in the Petri dish (Fig. S2c). Clear inhibition zones are visible on each spot, but with different diameters due to the lack of accuracy to measure exactly a quantity of 0.1 mg of CoNPs nanopowder added on each spot. To confirm the antimicrobial properties, antimicrobial tests against *E. coli*, *P. aeroginosa*, and *S. aureus* were also performed with CoNPs concentration of 0.001 mg/mL, 0.01 mg/mL, 0.1 mg/mL and 1 mg/mL (See main manuscript). Figure S2d shows the drop diffusion assay tests against E. coli for the 4 different CoNPs concentration, but similarly to SAE-AgNPs the antimicrobial test results were not fully reproducible due to the metallic nature of the CoNPs and the absence of organic coating on the surface that would have generated a colloidal solution.

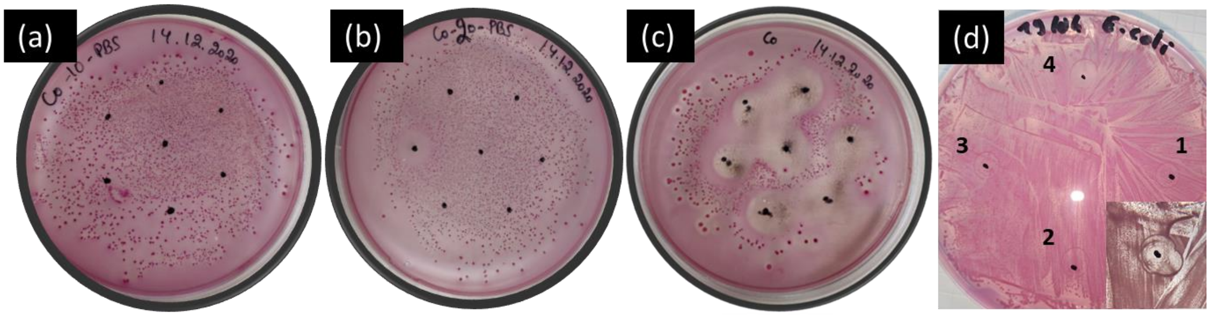

Figure S2. Antimicrobial activities of cobalt metal nanoparticles (CoNPs) using drop diffusion assay against *Escherichia coli*, (a) with CoNPs concentration of 0.2 mg/mL, (b) with CoNPs concentration of 0.4 mg/mL, (c) by dropping 0.1 mg of CoNPs powder on the 7 spots, (d) with CoNPs concentrations of 0.001, 0.01, 0.1 and 1 mg/mL that correspond to places marked 1 to 4, respectively. The inset shows higher magnification of a inhibition area measured for CoNPs concentration of 1 mg/mL.

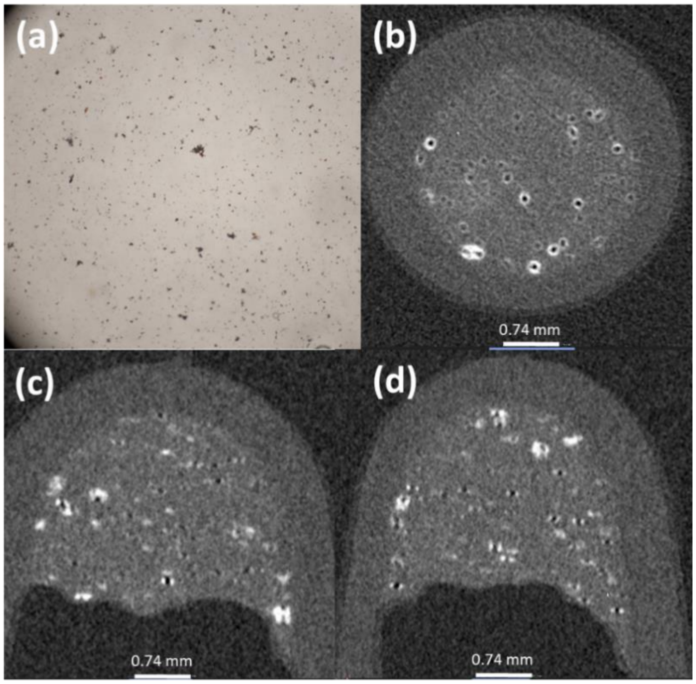

Figure S3. (a) Optical microscope photo of Aloe Vera gel mixed with cobalt metal nanoparticles (1wt%), (b), (c) and (d) X-ray tomography of Aloe Vera gel mixed with CoNPs (1wt%), top view, front view and right side view respectively.

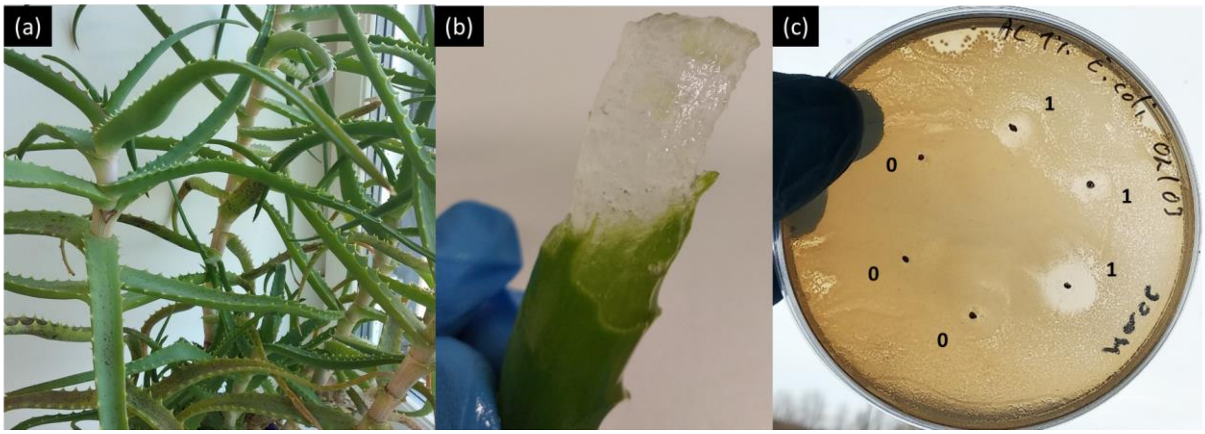

Figure S4. (a) *Aloe vera arborescent*, (b) fresh *Aloe vera* pulp from leaf, (c) Antimicrobial activities of cobalt metal nanoparticles mixed with fresh Aloe Vera gel against *Escherichia coli*,

the places marked 0 and 1 correspond to pure Aloe Vera gel and CoNPs weight ratio of 1wt%, respectively.
